# Myofibroblastoma of the breast: two case reports and literature review

**DOI:** 10.1093/jscr/rjab133

**Published:** 2021-04-22

**Authors:** Lorenzo Scardina, Gianluca Franceschini, Ersilia Biondi, Alba Di Leone, Alejandro Martin Sanchez, Sabatino D'Archi, Elena Jane Mason, Giuseppe Angelico, Angela Santoro, Antonino Mulè, Riccardo Masetti

**Affiliations:** 1 Centro Integrato di Senologia, Dipartimento Scienze della Salute della Donna, del Bambino e di Sanità Pubblica, Fondazione Policlinico Universitario Agostino Gemelli IRCCS, Rome, Italy; 2 Unità di Gineco-patologia e Patologia Mammaria, Dipartimento Scienze della Salute della Donna, del Bambino e di Sanità Pubblica, Fondazione Policlinico Universitario Agostino Gemelli IRCCS, Rome, Italy

## Abstract

Myofibroblastoma (MFB) is a relatively rare tumor of the breast parenchyma, which belongs to the family of the ‘benign stromal tumor of the breast’. Two cases of MFB of the breast are described. Radiological imaging is nonspecific in MFB, and pathological examination of needle biopsy or surgically resected specimen is necessary for the diagnosis. Surgery is recommended and considered curative without additional treatment; however, patients should be followed-up.

## INTRODUCTION

Myofibroblastoma (MFB) is a relatively rare tumor of the breast parenchyma, which belongs to the family of the ‘benign stromal tumor of the breast’. It is typically a bland-looking spindle cell tumor exhibiting morphological, immunohistochemical and ultrastructural features of both fibroblasts and myofibroblasts differentiation [1]. The prevalence is unknown, but probably accounts for <1% of all breast neoplasms with an age distribution ranging from 25 to 87 [2–4]. MFB is usually identified as an asymptomatic, slow-growing mass, well-defined, nontender and mobile on clinical examination [2–4]. MFB can arise in extra-mammary sites, along the milk-line and have been reported in the literature different sites including axilla, tonsil, lung, rectum, meninges, prostate, parotid gland and tongue [2–4].

Tumors with similar morphological and immunohistochemical features have also been reported in soft tissues and vagina with the term mammary-type MFB [3–6]. However, mammary and extra-mammary MFBs harbor the same chromosomal aberration, namely 13q14 deletion, detected by FISH analyses [3–5]. Similar molecular findings have also been observed in other benign tumors such as spindle cell lipoma of soft tissues and cellular angiofibroma of the lower female genital tract, supporting the hypothesis that all these myofibroblastic neoplasms are likely to arise from a common precursor cell [7].

We herein report the clinical, radiological and pathological findings observed in two MFB cases treated in the Division of Breast Surgery.

## CASE PRESENTATIONS

### Case 1

**Figure 1:**
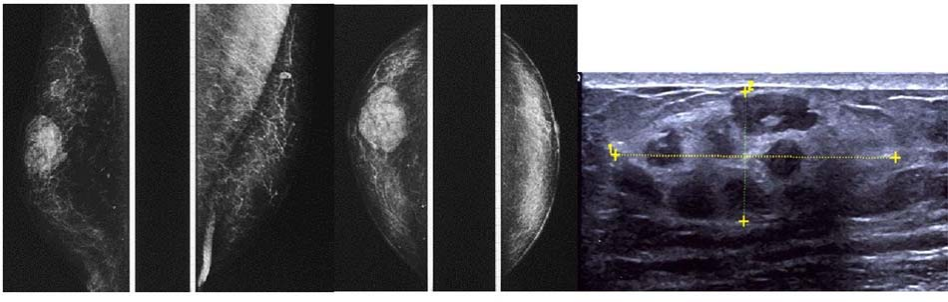
The diagnostic bilateral mammography in mediolateral oblique and craniocaudal views showed a well-circumscribed, oval, high density lesion in the right upper retroareolar region with no microcalcifications. Ultrasonography revealed a 36 mm-sized well-circumscribed mass of the right breast.

**Figure 2:**
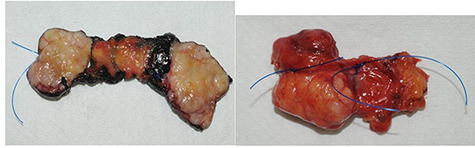
The macroscopic pathological examination showed a 36 mm-sized well-defined margins lesion

**Figure 3:**
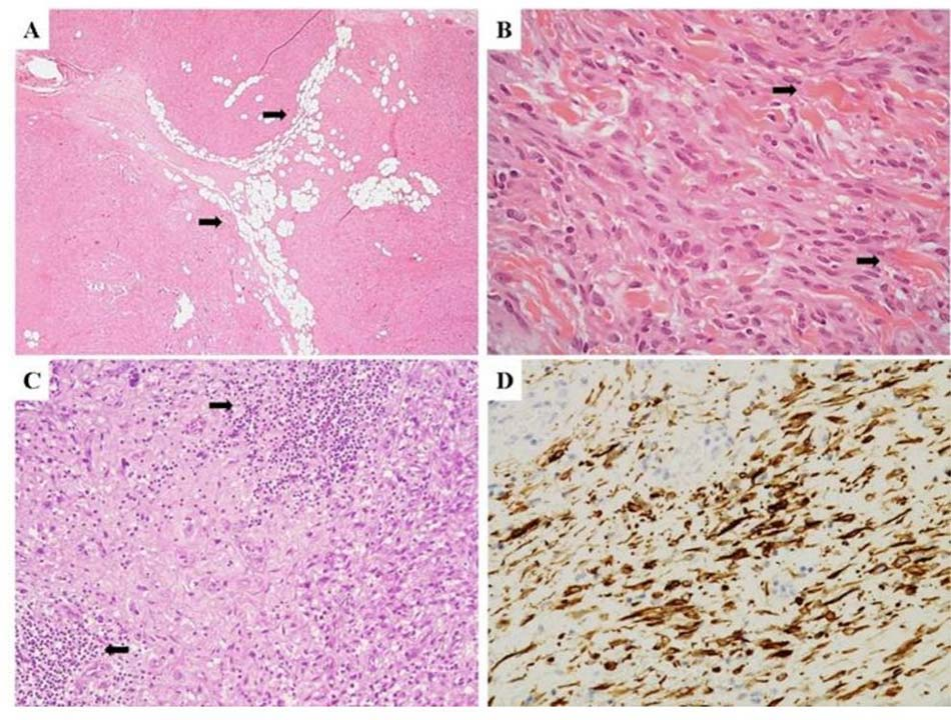
(**A**) Morphological features of Case 1, demonstrating a solid, cellular neoplasm with a fascicular growth pattern; interspersed, mature adipocytes were observed within the tumor stroma (arrows). (**B**) Neoplastic cells showed a spindled morphology with interspersed thick collagen bundles, a key diagnostic feature of MFB (arrows). (**C**) A densely collagenous stroma with interspersed lymphoid aggregates (arrows) was observed in some neoplastic areas. (**D**) Diffuse immunoreactivity for desmin confirmed the fibro-myofibroblastic lineage of neoplastic cells

In 2012, an 80-year-old man with a retroareolar mass on his right breast presented in our Breast Unit. He had no family history of breast or ovarian cancer, and he was affected by systemic arterial hypertension and diabetes mellitus type 2 on drug therapy. Clinical examination revealed no skin changes or nipple retraction, and there were not lymphadenopathies at the axillary sites. The patient reported the onset of a solid, painless, freely mobile lump, located in the upper retroareolar site of his right breast. The diagnostic bilateral mammography and ultrasonography revealed a 36 mm-sized well-circumscribed mass with no microcalcifications (BIRADS 3). Ultrasound (US) image showed increased vascularity of the mass without specific features suggestive of malignancy (Fig. 1). US-guided needle core biopsy was performed and revealed a neoplastic proliferation composed of spindle cells without cytological atypia nor mitotic activity. The patient underwent surgical lumpectomy under general anesthesia and the nodular mass was sent for pathological examination (Fig. 2). The pathological examination of the resected specimen confirmed the benign nature of the neoplasm and the fibro-myofibroblastic lineage of tumor cells; therefore, a final diagnosis of MFB of the breast was made (Fig. 3).

### Case 2

**Figure 4:**
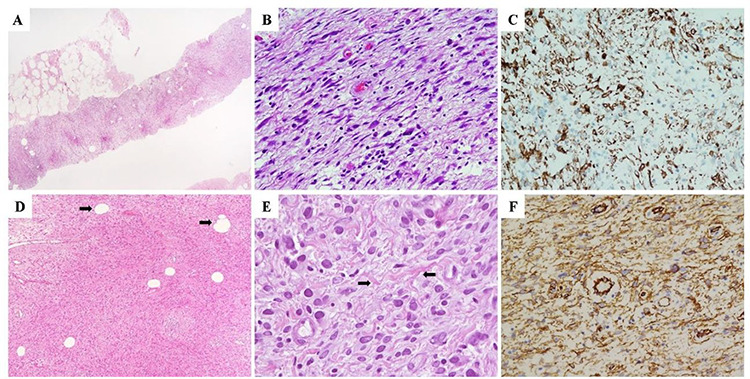
(**A**–**C**) Morphological and immunohistochemical features of the needle core biopsy demonstrating a solid, cellular neoplasm composed of spindle-shaped cells without cytological atypia nor mitotic activity (A and B). (C) Neoplastic cells demonstrated diffuse immunohistochemistry positivity for desmin, which, with the negative stainings for smooth muscle actin, CD34, S100 and pan-cytokeratins (AE1-AE3), was highly suggestive for myofibroblastic differentiation. On the basis of the above-mentioned findings, a descriptive pathological diagnosis of ‘myofibroblastic neoplasm without atypical features’ (B3) was rendered. (**D**) Morphological and imunohistochemical features of the surgically resected specimen demonstrating a cellular, bland-looking, spindle cell neoplasm with a minoritary adipocytic component (arrows). (**E**) Neoplastic cells showed a marked morphological variability characterized by spindled, oval and epithelioid shapes; interspersed collagen bundles were also observed within neoplastic stroma (arrows). (**F**) Diffuse immunoreactivity for desmin, consistent with fibro-myofibroblastic differentiation of neoplastic cells was observed

In July 2020, a 59-year-old female presented a palpable, nontender, mobile mass on her right breast. She denied personal or family history of breast or ovarian cancer, and she was affected by systemic arterial hypertension. Subsequent diagnostic mammography revealed a 20 × 20 mm high-density ovoid mass already reported in a breast magnetic resonance imaging performed in 2017 (BIRADS 3). The needle core biopsy revealed a cellular neoplasm composed of bland-looking spindle cells with a fascicular growth. The patient underwent surgical lumpectomy of the mass under general anesthesia. Pathological examination of the resected tumor confirmed the myofibroblastic nature of neoplastic cells which showed no worrisome morphological features (mitotic activity, nuclear atypia and necrosis). Based on these findings, a final diagnosis of MFB of the breast was rendered (Fig. 4).

## DISCUSSION

MFB was first described in 1981 and was named by Wargotz *et al*. [8] in 1987, using a series of 16 cases in which 11 patients were men and the average age was 63 years. In 2001, a case of extramammary MFB was described by McMenamin and Fletcher [3]. Subsequently, few other cases of MFB arising in older men (between 60 and 70 years old) have been described in the literature, as spindle cell lesion of the male breast associated with gynaecomastia [4]. From early reports, MFB seemed to occur more frequently in men; however, subsequent reports have suggested that it does not have a sexual predilection [9–11]. Therefore, MFB could be confused with different benign lesions of the breast such as leiomyoma, hematoma, abscess, neurofibroma, lymphangioma and fibroadenoma, and also malignant tumors such as sarcoma, lymphoma, malignant fibrous histiocytoma, and phyllodes tumor. Radiological imaging is nonspecific in MFB and pathological examination of needle biopsy or surgically resected specimen is necessary for the diagnosis. However, several authors have recently underlined that MFB of the breast represent a heterogeneous group of tumors that may exhibit a wide spectrum of histological features including myxoedematous and lipomatous changes, deciduoid morphology, collagenized areas with pseudoangiomatous stromal hyperplasia, cellular areas with focal cytological atypia, cartilaginous differentiation and an infiltrative growth pattern [4, 5, 12, 13]. All the above-mentioned variants pose differential diagnostic problems with a wide variety of benign and malignant mammary spindle cell lesions. For this reason, needle core biopsy specimens may represent a diagnostic challenge, especially when dealing with unusual morphological variants. On the contrary, pathological examination of the resected specimen allows a more precise diagnosis in most cases. In our opinion, the absence of marked cytologic atypia, along with low mitotic count and absence of atypical mitoses and necrosis, is helpful diagnostic clues to exclude malignancy.

There has been no report in the literature regarding diagnostic treatment modalities, but we strongly recommend breast US sonography, mammography and US-core needle biopsy to get a diagnosis before surgery.

In summary, MFB is a rare benign mesenchymal tumor of the breast. Surgery is recommended and considered curative without additional therapies such as radiation or hormonal therapies. Additionally, malignant transformation has not been reported yet. There is only one case in the literature of recurrence of MFB at the previous excisional site [4]. However, annual follow-up with ultrasonography and bilateral mammography (turned 50 years old) is recommended. Further studies with larger number of patients and longer follow-up are necessary to draw more validated conclusions.
